# Is metabolic syndrome a risk factor in hepatectomy? A meta-analysis with subgroup analysis for histologically confirmed hepatic manifestations

**DOI:** 10.1186/s12916-022-02239-x

**Published:** 2022-01-28

**Authors:** Anastasia Murtha-Lemekhova, Juri Fuchs, Svenja Feiler, Erik Schulz, Miriam Teroerde, Eva Kalkum, Rosa Klotz, Adrian Billeter, Pascal Probst, Katrin Hoffmann

**Affiliations:** 1 Department of General, Visceral, and Transplantation Surgery, Heidelberg University Hospital, Heidelberg, Germany; 2 The Study Center of the German Surgical Society (SDGC), Heidelberg University Hospital, Heidelberg, Germany; 3 Department of Surgery, Cantonal Hospital Thurgau, Frauenfeld, Switzerland

**Keywords:** Metabolic syndrome, liver surgery, post-hepatectomy liver failure, meta-analysis, NASH, NAFLD, MAFLD

## Abstract

**Background:**

Metabolic syndrome (MetS) is a risk factor in surgery. MetS can progress to metabolic (dysfunction)-associated fatty liver disease (MAFLD), a vast-growing etiology of primary liver tumors which are major indications for liver surgery. The aim of this meta-analysis was to investigate the impact of MetS on complications and long-term outcomes after hepatectomy.

**Methods:**

The protocol for this meta-analysis was registered at PROSPERO prior to data extraction. MEDLINE, Web of Science, and Cochrane Library were searched for publications on liver resections and MetS. Comparative studies were included. Outcomes encompassed postoperative complications, mortality, and long-term oncologic status. Data were pooled as odds ratio (OR) with a random-effects model. Risk of bias was assessed using the Quality in Prognostic Studies tool (QUIPS), and the certainty of the evidence was evaluated with GRADE. Subgroup analyses for patients with histopathologically confirmed non-alcoholic fatty liver disease (NAFLD) versus controls were performed.

**Results:**

The meta-analyses included fifteen comparative studies. Patients with MetS suffered significantly more overall complications (OR 1.55; 95% CI [1.05; 2.29]; *p*=0.03), major complications (OR 1.97 95% CI [1.13; 3.43]; *p*=0.02; *I*^2^=62%), postoperative hemorrhages (OR 1.76; 95% CI [1.23; 2.50]; *p*=0.01) and infections (OR 1.63; 95% CI [1.03; 2.57]; *p*=0.04). There were no significant differences in mortality, recurrence, 1- or 5-year overall or recurrence-free survivals. Patients with histologically confirmed NAFLD did not have significantly more overall complications; however, PHLF rates were increased (OR 4.87; 95% CI [1.22; 19.47]; *p*=0.04). Recurrence and survival outcomes did not differ significantly. The certainty of the evidence for each outcome ranged from low to very low.

**Conclusion:**

Patients with MetS that undergo liver surgery suffer more complications, such as postoperative hemorrhage and infection but not liver-specific complications—PHLF and biliary leakage. Histologically confirmed NAFLD is associated with significantly higher PHLF rates, yet, survivals of these patients are similar to patients without the MetS. Further studies should focus on identifying the tipping point for increased risk in patients with MetS-associated liver disease, as well as reliable markers of MAFLD stages and early markers of PHLF.

**Trial registration:**

PROSPERO Nr: CRD42021253768

**Supplementary Information:**

The online version contains supplementary material available at 10.1186/s12916-022-02239-x.

## Background

Metabolic syndrome (MetS) is a growing epidemic, marked by diabetes and obesity, that not only leads to increased rates of heart, vascular and gastrointestinal diseases, but presents a major risk factor in surgery [1, 2]. In addition to increasing as the cause of hepatocellular carcinoma (HCC), MetS is associated with gastrointestinal and biliary tree cancers, thus being accountable for the rising indications in hepatobiliary surgery [3, 4]. Non-alcoholic fatty liver disease (NAFLD) and non-alcoholic steatohepatitis (NASH) are considered the hepatic manifestation of the MetS and are now summarized as the metabolic (dysfunction) associated fatty liver disease (MAFLD) [5]. This condition can lead to HCC without the conventional sequence of steatosis, steatohepatitis, fibrosis, cirrhosis, and, ultimately, HCC [6].

With indications for hepatobiliary surgery expanding, investigation of risk and predictive factors for post-hepatectomy liver failure (PHLF), adequate postoperative liver regeneration, and other surgery-related outcomes intensifies in clinical importance [7, 8]. An accepted dogma of liver surgery dictates that for sufficient regeneration after hepatectomy, the future liver remnant (FLR) requires the volume of at least 20% from the initial organ. In case the liver parenchyma is altered due to chemotherapy or steatosis, e.g., due to MAFLD or alcohol overindulgence, at least 30% of the volume is required for the liver to sufficiently regenerate after resection [9].

As MetS is not only viewed as a patient-related risk factor for surgery but also as a risk factor for liver-specific complications in patients requiring liver surgery, a closer evaluation is warranted. The aim of this meta-analysis is to evaluate the MetS as a risk factor for post-hepatectomy complications as well as a predictor of adverse oncological outcome.

## Methods

The systematic review is reported according with the current PRISMA guidelines [10] and follows the recommendations of the Cochrane Handbook for Systematic Reviews and Interventions [11]. The protocol for this systematic review and meta-analysis was registered at PROSPERO prior to data extraction (CRD42021253768) [12].

The literature search was conducted in accordance with the recent recommendations [13]. The aim of the search was to identify all publications on liver resections in context of metabolic syndrome in humans. The search strategy is provided in Supplementary material. The searches were performed using MEDLINE via PubMed, Web of Science, and Cochrane Library. The last search was completed on August 27, 2021. Neither language nor publication date were restricted. Additionally, a hand search through references of included studies was performed.

Comparative study methodology was sought after with no restriction on prospective or retrospective design or blinding. Comments, editorials, meeting abstracts, correspondence, and reviews were excluded. The screening of titles and abstracts was performed by two independent reviewers (AML and JF). All disagreements were resolved through discussion and consultation with the third reviewer (KH). Full-text review was performed independently by the same reviewers, after which all disagreements were once again resolved through discussion and consultation with the same third reviewer.

Data of included studies was extracted by two reviewers (AML and JF) independently using a standardized form composed prior to data extraction and adjusted based on the first two data extractions. Extracted data for each publication encompassed: title, authors, year of publication, country of publication, journal, source of funding, study methodology, cohorts’ characteristics, interventions, and clinical outcomes (complication rates and gradings for post-hepatectomy liver failure, biliary leakage, bleeding, infections, Clavien-Dindo grades, mortality, recurrence, overall and recurrence-free survivals).

Meta-analyses were performed with R (R version 4.0.3 using packages “metafor,” “meta,” and “ggplot2”). Forest plots present effect estimates. A random-effects model was utilized for all outcomes due to the heterogenic methodological and clinical framework of included studies [13]. Statistical heterogeneity was evaluated using the *I*^2^ statistics. An *I*^2^ value below 25% indicated low heterogeneity, while over 75% was considered high. Odds ratios and 95% confidence intervals for dichotomous endpoints were pooled with the Mantel-Haenszel method

The methodological quality of included studies was performed using QUIPS [14]. Detailed information about assessed qualities is provided in Table 1. The certainty of the evidence was assessed using GRADE [15, 16].

**Table 1:** QUIPS

Study	Study participation	Study attrition	Prognostic factor measurement	Outcome measurement	Study confounding	Statistical analysis and reporting
Koh 2019	Moderate	High	Moderate	Low	Moderate	Moderate
Nishioka 2016	Moderate	High	Low	Low	Moderate	Moderate
Billeter 2020	Moderate	High	Moderate	Low	Moderate	Moderate
Hobeika 2019	Moderate	Moderate	Moderate	Low	Moderate	Moderate
Bhazani 2012	Moderate	High	Moderate	Low	Moderate	Moderate
Pais 2017	Moderate	Moderate	Moderate	Low	Moderate	Moderate
Tian 2018	Moderate	High	Moderate	Low	Moderate	Moderate
Tian 2020	Moderate	High	High	Low	Low	Moderate
Yang 2020	Moderate	High	Moderate	Low	Low	Moderate
Yoshida 2015	Moderate	High	Moderate	Low	Moderate	Moderate
Le Bien 2012	Moderate	High	Moderate	Low	High	Moderate
Jung 2020	Moderate	High	Moderate	Low	Moderate	Moderate
Wakai 2011	Moderate	High	Moderate	Low	Moderate	Moderate
Conci 2021	Moderate	Moderate	Low	Low	Moderate	Moderate
Fagenson 2020	Moderate	Moderate	Moderate	Low	Moderate	Moderate

Based on the result of the full-text examination, the following endpoints could be evaluated in the quantitative analysis: overall complications, minor and major complications, PHLF, postoperative infections, postoperative biliary leakage and hemorrhage, perioperative and 90-day mortalities, recurrence, 1- and 5-year overall survivals (OS), and 1- and 5-year recurrence-free survivals (RFS).

## Results

**Fig. 1:**
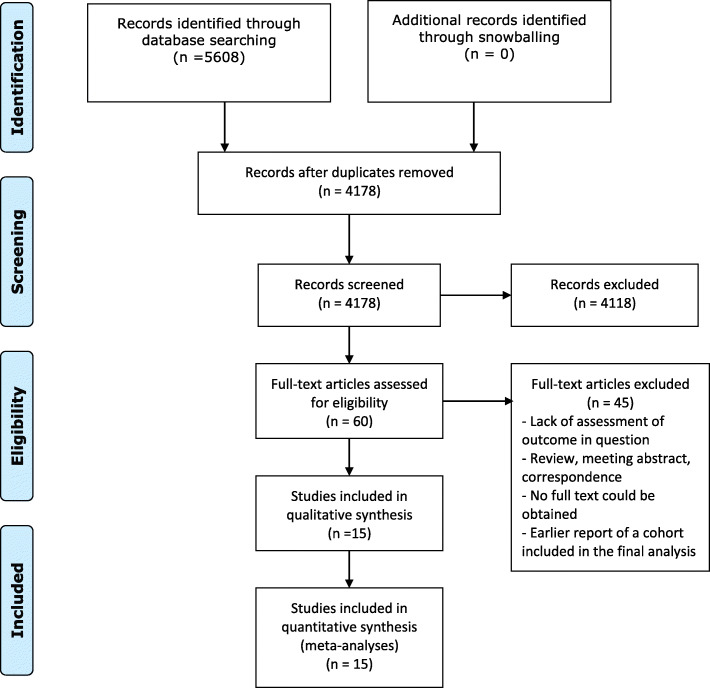
Flowchart of the selection process. The flow diagram depicts the steps of the systematic review process

The search identified 4178 potentially relevant publications after exclusion of duplicates. After screening of titles and abstracts, 60 full texts were further evaluated for inclusion. Inclusion criteria were fulfilled by fifteen articles which were then included into qualitative and quantitative analyses (Fig. 1). Although most studies described patients with HCC [17–26], two studies [27, 28] reported on patients undergoing hepatectomy for intrahepatic cholangiocarcinoma and another three on patients with various benign and malignant liver tumors [29–31]. Table 2 provides an overview of core data extracted from each study. Only two studies described patients undergoing major hepatectomy while others had predominantly minor hepatectomy cohorts [30, 31]. Due to different endpoints used by both studies, no quantitative analysis could be performed for major hepatectomy cohorts.

**Table 2:** Overview of included publications

Publication	Methodology	Definition of MetS/NAFLD used	Included in subgroup analysis for histologically proven NAFLD	Cohorts		Indications		Hepatectomy	
				MetS (***n***)	Control (***n***)	MetS ***n*** (%)	Control ***n*** (%)	MetS ***n*** (%)	Control ***n*** (%)
Koh 2019	Retrospective, single center	NAFLD: NASH Clinical Research Network criteria [32]	Yes	152	844	HCC 152	HCC 844	Major 21 (14%) Minor 131 (86%)	Major 238 (28%) Minor 606 (75%)
Vigano 2015	Retrospective, multicenter, matched	MetS: National Cholesterol Education Program’s Adult Treatment Panel III report [33]	No	96	96	HCC 96	HCC 96	Major 41 (43%) Minor 55 (57%)	Major 41 (43%) Minor 55 (57%)
Nishioka 2016	Retrospective, single center	MetS: diabetes, HT and dyslipidemia	No	14	15	ihCC 14	ihCC 13	Major 13 (93%) Minor 1 (7%)	Major 13 (100%) Minor 0
Billeter 2020	Retrospective, single center, matched	NAFLD: SAF score [34]	Yes	34	54	HCC 34	HCC 54	Major 19 (56%) Minor 15 (44%)	Major 7 (13%) Minor 47 (87%)
Hobeika 2019	Retrospective, single center	MetS: ≥3 criteria—central obesity, dyslipidemia, DM2/glucose intolerance, HT. NAFLD: NASH Clinical Research Network Scoring System [32]	Yes	40	75	ihCC 40	ihCC 75	Major 28 (70%) Minor 12 (30%)	Major 63 (84%) Minor 12 (16%)
Bhayani 2012	Retrospective, database	MetS: BMI≥30 kg/m^2^, diabetes and HT	No	256	3717	Metastasis 113 (44%) HCC 75 (29%) ihCC 9 (4%) Other malignancy 18 (7%) Benign lesions 14 (5%)	Metastasis 1717 (46%) HCC 596 (16%) ihCC 130 (4%) Other malignancy 253 (7%) Benign lesions 441 (7%)	Major 86 (34%) Minor 170 (66%)	Major 1546 (42%) Minor 2171 (58%)
Pais 2017	Retrospective, multicenter	NAFLD: metabolic risk factors (overweight or obesity defined as BMI ≥25 kg/m^2^, DM2, HT, dyslipidemia), and absence of other causes of liver disease	No	39	284	HCC 39	HCC 284	Major 19 (49%) Minor 20 (51%)	Major 108 (38%) Minor 176 (62%)
Tian 2018	Retrospective, single center	MetS: central obesity (waist circumference ≥ 90 cm for men, ≥ 80 cm for women) plus 2 of the following: elevated triglycerides (≥ 150 mg/dL or specific treatment), reduced HDL cholesterol (< 40 mg/dL in males and < 50 mg/dL in females or specific treatment), elevated BP (systolic BP ≥ 130 or diastolic BP ≥ 85 mmHg or treatment for HT), elevated fasting plasma glucose ≥ 100 mg/dL or DM2	No	81	1154	HCC 81	HCC 1154	Major 9 (11%) Minor 72 (89%)	Major 204 (18%) Minor 950 (92%)
Tian 2020	Retrospective, single center, matched	MetS: central obesity (waist circumference with ethnicity-specific values) and any two: reduced HDL (< 40 mg/dL in males and < 50 mg/dL in females or specific treatment), elevated triglycerides (≥ 150 mg/dL or specific treatment), elevated fasting plasma glucose ≥ 100 mg/dL or DM2, elevated BP (systolic BP ≥ 130 or diastolic BP ≥ 85 mmHg or treatment for HT)	No	74	74	HCC 74	HCC 74	Major 4 (5%) Minor 70 (95%)	Major 2 (3%) Minor 72 (97%)
Yang 2020	Retrospective, multicenter, matched	NAFLD is defined as MetS (overweight or obesity, DM2, HT, and dyslipidemia), consistent US features of fatty liver, and/or past or present histological features of hepatic fatty infiltration with an alcohol intake < 30 g/day	No	89	89	HCC 89	HCC 89	Major 32 (36%) Minor 57 (64%)	Major 27 (30%) Minor 62 (70%)
Yoshida 2015	Retrospective, single center	MetS: ≥3 of the following: central obesity (waist circumference ≥ 90 cm in men and ≥80 cm in women, BMI ≥ 28 kg/m2, dyslipidemia (triglycerides ≥150 mg/dL or HDL ≥40 mg/dL in men, ≥50 mg/dL in women), HT (BP ≥130/85 mmHg), and diabetes mellitus (fasting glucose [100 mg/dL)	No	35	114	HCC 35	HCC 114	Major 3 (9%) Minor 32 (91%)	Major 8 (7%) Minor 106 (93%)
Le Bian 2012	Retrospective, single center	MetS: ≥3 of the following: overweight or obesity (BMI > 25 kg/m^2^), DM (defined as fasting plasma glucose > 5.5 mmol/L), HT (BP > 130/85 mmHg), and dyslipidemia (triglycerides ≥ 1.7 mmol/l and/or HDL < 1 mmol/L in males or < 1.3 mmol/L in females).	No	30	121	Primary 10 (33%) Metastasis 17 (57%) Benign 3 (10%)	Primary 29 (24%) Metastasis 59 (49%) Benign 33 (27%)	Major 30	Major 121
Jung 2020	Retrospective, single center, matched	NAFLD criteria of the American Association for the Study of Liver Disease [33]	Yes	32	32	HCC 32	HCC 32	Major 24 (75%) Minor 8 (25%)	Major 18 (56%) Minor 14 (44%)
Wakai 2011	Retrospective, single center	NAFLD: NASH Clinical Research Network criteria [32]	Yes	17	208	HCC 17	HCC 208	Major 8 (47%) Minor 9 (53%)	Major 59 (28%) Minor 149 (72%)
Conci 2021	Retrospective, multicenter, matched	MAFLD: hepatic steatosis (imaging, blood scores/markers, or histology) and overweight (BMI ≥25 kg/m^2^ in Caucasians or ≥23 in Asians) or DM2 or normal weight in the presence of two or more metabolic abnormalities (high waist circumference, HT, elevated triglycerides or cholesterol, insulin resistance or prediabetes, and high level of plasma c-reactive protein).	No	85	255	HCC 85	HCC 255	Major 18 (21%) Minor 67 (79%)	Major 44 (17%) Minor 211 (83%)
Fagenson 2020	Retrospective, database, matched	MetS: 3 elements obesity (BMI > 30 kg/m2, HT, and diabetes mellitus)	No	863	863	Primary 261 (30%) Metastasis 457 (53%) Benign 145 (17%)	Primary 258 (30%) Metastasis 462 (53%) Benign 143 (17%)	Major 863	Major 863

*BP* blood pressure, *DM2* diabetes mellitus type 2, *HCC* hepatocellular carcinoma, *HDL* high-density lipoprotein cholesterol, *HT* hypertension, *MAFLD* metabolically associated fatty liver disease, *MetS* metabolic syndrome, *NAFLD* non-alcoholic fatty liver disease, *NASH* non-alcoholic steatohepatitis *US* ultrasound

### Critical appraisal of included studies

The risk of bias assessment was performed using the tool for Quality in Prognostic Studies (QUIPS) and included assessment of six main domains: study participation, study attrition, prognostic factor measurement, outcome measurement, study confounding, and statistical analysis and reporting. Table 1 provides an overview of the assessment. Overall, included studies had a moderate risk of bias.

### Complications

**Fig. 2:**
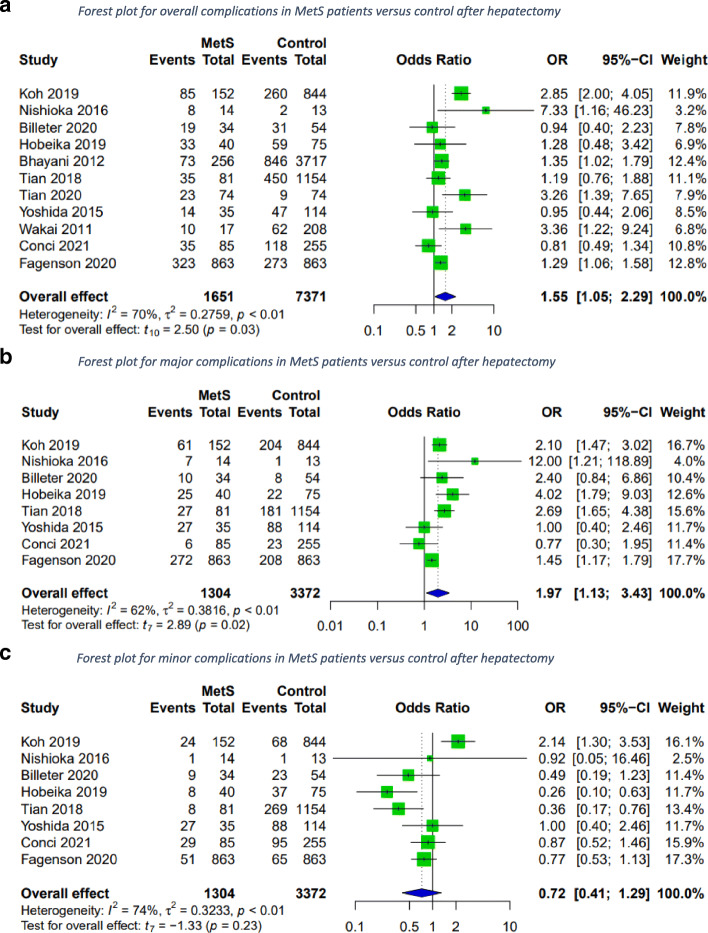
Forest plots for overall, major and minor complications in MetS patients versus control after hepatectomy. A random-effects model was utilized for all outcomes due to heterogenic methodological and clinical framework of included studies. Statistical heterogeneity was evaluated using the *I*^*2*^ statistics. An *I*^*2*^ value below 25% indicated low heterogeneity, while over 75% was considered high

Overall complications were assessed based on data from eleven studies [17, 19, 21–23, 25–29, 31]. Data on major and minor complications was available in eight [17, 19, 22, 25–28, 31]. Overall, patients with the MetS suffered significantly more overall complications compared to control. There was also a significant increase in major complications for patients with MetS (Fig. 2).

**Fig. 3:**
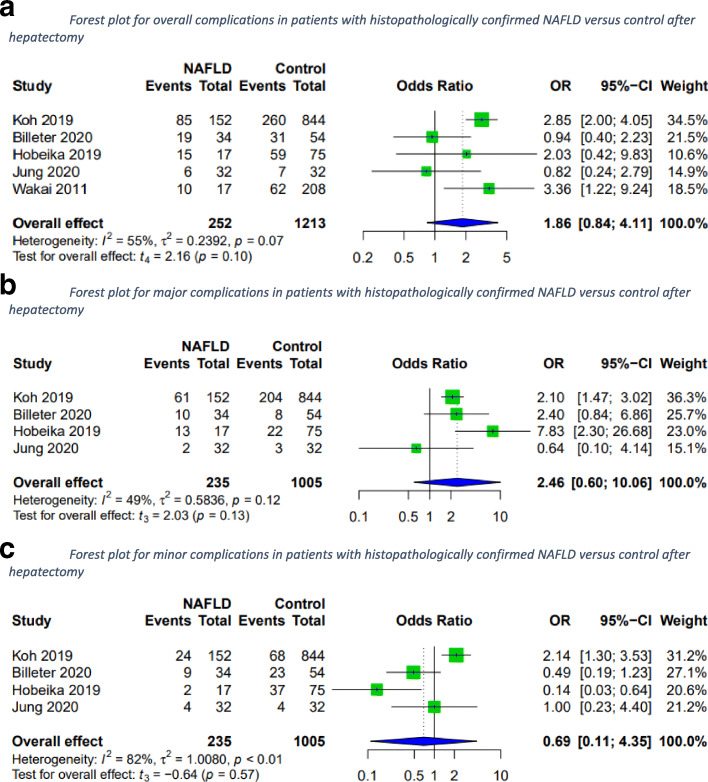
Forest plots for overall, major and minor complications in patients with histopathologically confirmed NAFLD versus control after hepatectomy. A random-effects model was utilized for all outcomes due to the heterogenic methodological and clinical framework of included studies. Statistical heterogeneity was evaluated using the *I*^*2*^ statistics. An *I*^*2*^ value below 25% indicated low heterogeneity, while over 75% was considered high

In a subgroup analysis of patients with histopathologically confirmed NAFLD versus control, the two groups did not vary significantly in overall complications. No difference was observed for major or minor complications either (Fig. 3).

### Liver-specific complications

**Fig. 4:**
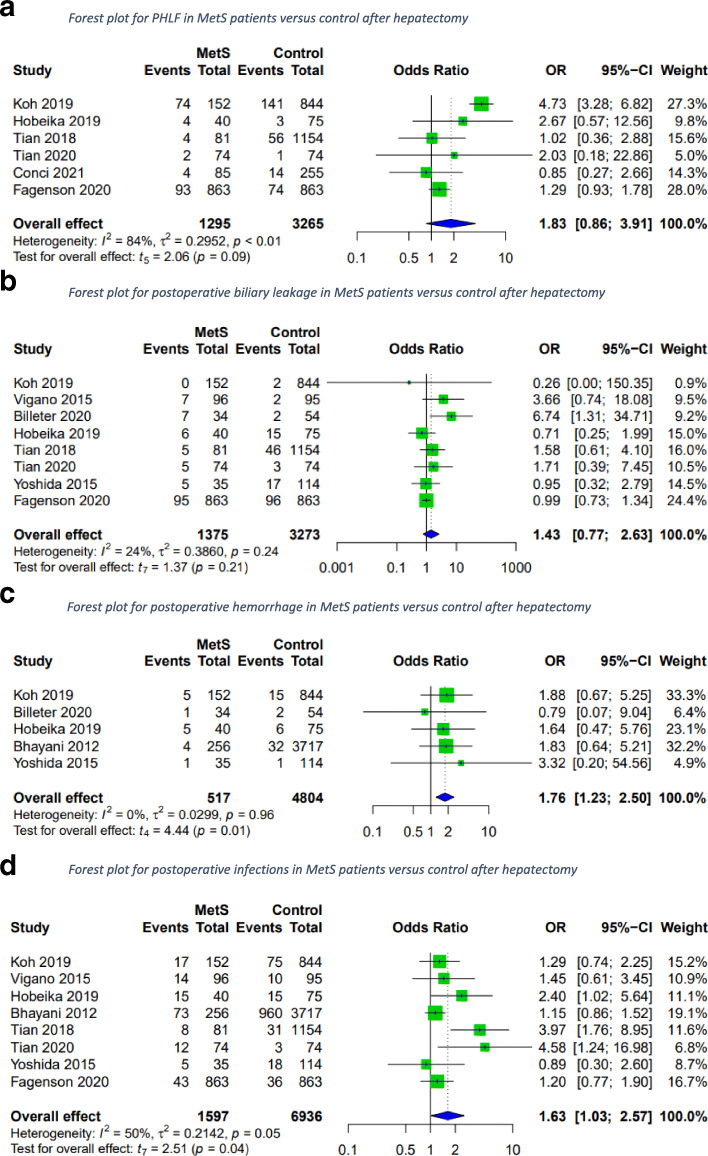
Forest plots for liver-specific complications in MetS patients versus control after hepatectomy. A random-effects model was utilized for all outcomes due to the heterogenic methodological and clinical framework of included studies. Statistical heterogeneity was evaluated using the *I*2 statistics. An *I*^*2*^ value below 25% indicated low heterogeneity, while over 75% was considered high

Liver-specific complications after hepatectomy for patients with the MetS versus those without were assessed. PHLF rates, biliary leakages, and postoperative infections did not differ between groups; however, postoperative hemorrhage occurred significantly more frequently in MetS patients (Fig. 4).

**Fig. 5:**
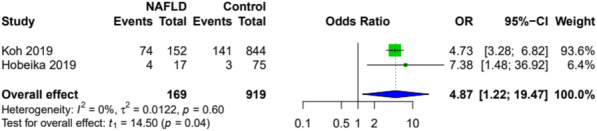
Forest plot for PHLF in patients with histopathologically confirmed NAFLD versus control after hepatectomy. A random-effects model was utilized for all outcomes due to the heterogenic methodological and clinical framework of included studies. Statistical heterogeneity was evaluated using the *I*^*2*^ statistics. An *I*^*2*^ value below 25% indicated low heterogeneity, while over 75% was considered high

In patients with histopathologically confirmed NAFLD, PHLF occurred more frequently than in the control group (Fig. 5), while postoperative biliary leakage, infection and hemorrhage were comparable to the control group (OR 1.21; 95 CI [0.01; 178.13]; *p*=0.88; *I*^2^=58%, OR 2.17; 95% CI [0.00; 5413.25]; *p*=0.43; *I*^2^=74%, and OR 2.08; 95% CI [0.49; 8.85]; *p*=0.16; *I*^2^=0%). Postoperative biliary leakage and hemorrhage were reported in three studies, while only two provided data on postoperative infections.

### Mortality

**Fig. 6:**
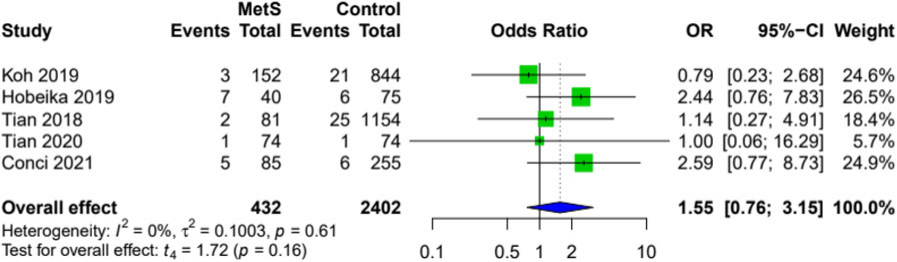
Forest plot for 90-day mortality in MetS patients versus control after hepatectomy. A random-effects model was utilized for all outcomes due to the heterogenic methodological and clinical framework of included studies. Statistical heterogeneity was evaluated using the *I*^*2*^ statistics. An *I*^*2*^ value below 25% indicated low heterogeneity, while over 75% was considered high

Perioperative mortality was assessed in two studies, which showed similar results in MetS patients and the control groups (OR 4.84; 95% CI [0.00; 41157788.03]; *p*=0.43; *I*^2^=62%). Ninety-day mortality was reported in five studies and showed only a minor difference in patients with and without MetS (Fig. 6).

Ninety-day mortality did not show a difference in the subgroup analysis for patients with histopathologically confirmed NAFLD versus control (OR 1.90; 95% CI [0.00; 179526.98]; *p*=0.61; *I*^2^=74%). However, only two studies reported this outcome. Perioperative mortality could not be analyzed for the subgroup due to insufficient data.

### Oncological outcomes

**Fig. 7:**
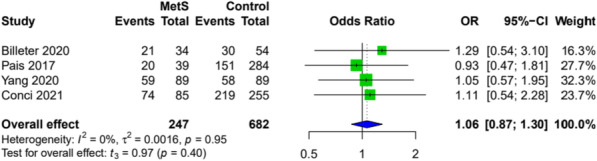
Forest plot for tumor recurrence in MetS patients versus control after hepatectomy. A random-effects model was utilized for all outcomes due to the heterogenic methodological and clinical framework of included studies. Statistical heterogeneity was evaluated using the *I*^*2*^ statistics. An *I*^*2*^ value below 25% indicated low heterogeneity, while over 75% was considered high

Recurrence rates were similar in patients with and without the MetS. Three studies provided sufficient data for the outcome (Fig. 7).

For patients with histopathologically confirmed NAFLD versus control, recurrence rates did not show significant differences (OR 1.11; 95% CI [0.69; 1.79]; *p*=0.43; *I*^2^=0%). However, only three studies provided data for this endpoint.

One-year OS was reported in five publications and did not show significant differences between groups (OR 0.83; 95% CI [0.32; 2.14]; *p*=0.61; *I*^2^=39%). Six publications described 5-year OS rates, which did not show a significant difference between groups (OR 1.07; 95% CI [0.79; 1.45]; *p*=0.58; *I*^2^=8%). These differences were also insignificant in patients with histopathologically confirmed NAFLD versus control: 1-year OS OR 0.79; 95% [0.00; 93283.03]; *p*=0.84; *I*^2^=80%, 5-year OS OR 1.39 [0.85; 2.26]; *p*=0.10; *I*^2^=0%. These overall effects estimated were based on data from two and three studies, respectively.

One-year RFS was similar in patients with and without the MetS (OR 0.95; 95% CI [0.47; 1.92]; *p*=0.84; *I*^2^=0%), as was the 5-year RFS (OR 1.20; 95% CI [0.91; 1.59]; *p*=0.14; *I*^2^=0%). These were also the effects seen in patients with histopathologically confirmed NAFLD versus control (1-year RFS OR 0.96; 95% CI [0.00; 920.79]; *p*=0.95; *I*^2^=19% and 5-year RFS OR 1.23; 95% CI [0.76; 1.99]; *p*=0.21; *I*^2^=0%)

### Certainty of evidence (GRADE)

A rating of certainty of evidence was made with the grading of recommendations assessment, development, and evaluation (GRADE) approach (Tables 3 and 4). An overview for the outcomes of the main analysis and for the two subgroup analyses are provided in Tables 3 and 4. The outcomes of the main analysis ranged from very low to low largely due to study designs of included studies (retrospective) and study biases. The certainty of the evidence for outcomes for histologically confirmed NAFLD versus control also ranged from low to very low due to study design, risk of bias, and imprecision. Due to a lack of studies, perioperative mortality could not be evaluated.

**Table 3:** Certainty of the evidence for outcomes of the main analysis

Outcome	№ of included studies	Certainty of the evidence (GRADE)	Relative effect (95% CI)
Overall complications	11 (retrospective)	⨁⨁◯◯ LOW	OR 1.55 [1.05; 2.29]
Major complications	8 (retrospective)	⨁⨁◯◯ LOW	OR 1.97 [1.13; 3.43]
Minor complications	8 (retrospective)	⨁◯◯◯ VERY LOW	OR 0.72 [0.41; 1.29]
PHLF	6 (retrospective)	⨁◯◯◯ VERY LOW	OR 1.83 [0.86; 3.91]
Postoperative biliary leakage	8 (retrospective)	⨁⨁◯◯ LOW	OR 1.43 [0.77; 2.63]
Postoperative hemorrhage	5 (retrospective)	⨁⨁◯◯ LOW	OR 1.76 [1.23; 2.50]
Postoperative infection	8 (retrospective)	⨁⨁◯◯ LOW	OR 1.63 [1.03; 2.57]
Perioperative mortality	2 (retrospective)	⨁◯◯◯ VERY LOW	OR 4.84 [0.00; 41157788.03]
90-days mortality	5 (retrospective)	⨁⨁◯◯ LOW	OR 1.55 [0.76; 3.15]
1-year overall survival	5 (retrospective)	⨁⨁◯◯ LOW	OR 0.83 [0.32; 2.14]
5-years overall survival	6 (retrospective)	⨁⨁◯◯ LOW	OR 1.07 [0.79; 1.45]
Recurrence	4 (retrospective)	⨁⨁◯◯ LOW	OR 1.06 [0.87; 1.30]
1-year recurrence-free survival	4 (retrospective)	⨁⨁◯◯ LOW	OR 0.95 [0.47; 1.92]
5-year recurrence-free survival	5 (retrospective)	⨁⨁◯◯ LOW	OR 1.20 [0.91; 1.59]

*CI* confidence interval, *OR* odds ratio

**Table 4:** Certainty of evidence for outcomes of the subgroup analysis for histologically confirmed NAFLD versus control

Outcome	№ of included studies	Certainty of the evidence (GRADE)	Relative effect (95% CI)
Overall complications	5 (retrospective)	⨁⨁◯◯ LOW	OR 1.86 [0.84; 4.11]
Major complications	4 (retrospective)	⨁⨁◯◯ LOW	OR 2.46 [0.60; 10.06]
Minor complications	4 (retrospective)	⨁◯◯◯ VERY LOW	OR 0.69 [0.11; 4.35]
PHLF	2 (retrospective)	⨁⨁◯◯ LOW	OR 4.87 [1.22; 19.47]
Postoperative biliary leakage	3 (retrospective)	⨁◯◯◯ VERY LOW	OR 1.21 [0.01; 178.13]
Postoperative hemorrhage	3 (retrospective)	⨁⨁◯◯ LOW	OR 2.08 [0.49; 8.85]
Postoperative infection	2 (retrospective)	⨁◯◯◯ VERY LOW	OR 2.17 [0.00; 5413.25]
Perioperative mortality	0	NE	NE
90-days mortality	2 (retrospective)	⨁◯◯◯ VERY LOW	OR 1.90 [0.00; 179526.98]
1-year overall survival	2 (retrospective)	⨁◯◯◯ VERY LOW	OR 0.79 [0.00; 93283.03]
5-years overall survival	3 (retrospective)	⨁⨁◯◯ LOW	OR 1.39 [0.85; 2.26]
Recurrence	3 (retrospective)	⨁⨁◯◯ LOW	OR 1.11 [0.69; 1.79]
1-year recurrence-free survival	2 (retrospective)	⨁⨁◯◯ LOW	OR 0.96 [0.00; 920.79]
5-year recurrence-free survival	3 (retrospective)	⨁⨁◯◯ LOW	OR 1.23 [0.76; 1.99]

*CI* confidence interval, *OR* odds ratio, *NE* not evaluated

## Discussion

The global prevalence of the metabolic syndrome is estimated at a quarter of the world’s population. NAFLD affects more than 200 million people worldwide and approximately 20% progress further to NASH [35]. These conditions not only increase primary liver cancer incidences but are a major risk factor for other cancers, such as colorectal, which often metastasizes to the liver [3, 4]. With more than 1.9 million new cases of colorectal cancers per year, 70% with hepatic metastases, and 0.9 million annual new cases of liver cancer, liver surgery remains the preferred treatment for most hepatic lesions [36, 37].

The armamentarium of hepatobiliary surgery is continuously expanding, thus widening the indications for surgery as well [38, 39]. With constant evolution of the field, it is necessary to re-evaluate accepted dogmas with current treatment possibilities and test the boundaries of presumably known risk factors. Experienced hepatobiliary surgeons must be included in tumor boards and treatment discussions to offer more patients potentially curative options within the realm of current possibilities [40].

Despite numerous clinical scores for NAFLD/MAFLD, a reliable clinical assessment for patients with manifesting MAFLD is not yet available [41]. Liquid-based biopsies have varying reliability with most investigated factors associated with steatohepatitis or fibrosis, meaning already established NASH [41]. The need for adequate and reliable preoperative assessments is evident, to identify patients in different stages of MAFLD and, thus, structure individualized therapy plans. This need is also underlined by the results of this meta-analysis. Patients with the MetS were not at significantly higher risk for PHLF, while patients with histologically confirmed NAFLD were. The question remains, at what stage the risk of PHLF increase in patients suffering from the MetS. Although postoperative hemorrhage presents a risk in liver surgery, this has not previously been described as a specific complication for the MetS. However, the significant increase of postoperative bleeding is evident in this meta-analysis. As the liver is the major source of mature pro- and anticoagulative factors and the balance of these is upset after hepatectomies [42], investigating individual coagulation profiles of patients with and without the MetS subjected to liver surgery becomes essential.

The recurrence rates for resected tumors were similar in patients suffering from MAFLD versus other etiologies, indicating that this group too profits from surgery. Additionally, survival rates were similar to controls in the main and subgroup analyses, further solidifying this conclusion.

This is the first meta-analysis to investigate the effect of MetS on hepatic resection and chosen endpoints are of direct relevance for surgery and oncological assessment. The limitation of this meta-analysis is primarily rooted in the publications that met the criteria for inclusion. As most studies were retrospective and investigated unmatched cohorts, there is a risk of bias. Patients with the MetS and those with histopathologically confirmed NAFLD are considered poor candidates due to comorbidities and hepatic changes—this may lead to a selection bias. Additionally, included studies used different criteria for diagnosis of MetS—histological confirmation of MetS-associated changes in the liver, two or three criteria for MetS as defined by national or international societies. Definition of NAFLD also varied between studies with several studies utilizing the NASH Clinical Research Network criteria, some studies choosing SAF score and some opting for criteria proposed by the national association for the study of liver disease [32–34]. This inhomogeneity of definition harbors a potential bias and underlines the need for systematic studies in the area and consensus of definitions. Comparative controls also differed between groups: intrahepatic cholangiocarcinomas were compared in patients with and without the MetS, as were various benign and malignant lesions without further division. Patients with MetS-associated HCC were compared to various etiologies, hepatitis C- or B- -associated HCC, hepatitis without further subdivision, and alcohol-associated HCC. Thus, the overall control group consists of a clinically representative albeit heterogenic group with sufficiently high power. Although heterogeneity of included studies was generally acceptable and often low in subgroup analyses, these analyses were often based on three studies alone. This inevitably leads to a wider 95% CI and, thus, deficit of significance. Unarguably, more studies investigating different stages of MAFLD, from simple MetS to manifesting NASH will strengthen the certainty of evidence provided by this meta-analysis. Especially impact of metabolically associated steatohepatitis on outcomes after hepatectomy will provide invaluable insight.

More emphasis should be placed on reliable but non-invasive markers of MAFLD-stages. Although a liver biopsy can provide valuable information and is the current standard of assessment for NAFLD/NASH diagnosis, due to associated risks and limited clinical relevance in absence of a tumor burden, this intervention has lost its popularity [43]. Additionally, the biopsy only assesses a minuscule area of the liver, assuming validity in generalization. When a resection of a liver tumor is planned, a preoperatively diagnosed MAFLD would not lead to an alteration of the treatment plan, thus a biopsy could be combined with a resection, in which case a representative area can be obtained for histopathological assessment. A serum-based marker would present a superior test to assess the patient, as little infrastructure and less specialized personnel are required. Also, a serum-based marker could be offered to a larger population. Additionally, the need for an early and reliable PHLF marker is evident [8]. As PHLF remains the most feared complication after hepatectomy with rates reaching up to 30%, early detection is invaluable to provide patients with adequate care and evade lethal consequences [7]. Liver systems medicine approach and multidisciplinary consortia that place their emphasis on PHLF and MAFLD may unveil intimate knowledge on pathogenesis and suggest viable treatment options that are currently lacking (https://www.lisym.org/).

## Conclusion

The metabolic syndrome presents a risk factor in hepatic surgery with patients suffering significantly more complications. Yet, liver-specific complications, such as PHLF and biliary leakage rates are not increased. Patients with MetS are at higher risk for postoperative hemorrhage and infections – complications that can be managed through adjustment of the surgical technique and perioperative care. Improvement of the preoperative differentiation of MetS with and without hepatic manifestation is overdue, as patients with MetS-associated histological changes are at greater risk for PHLF. Developing prognostic markers for PHLF remains a priority. Despite higher PHLF rates, patients with MAFLD show similar 1- and 5-year overall and recurrence-free survivals, thus clearly benefiting from surgery as much as patients with liver malignancies of other etiologies.

## Supplementary Information

**Additional file 1.** Supplementary material.

## Data Availability

All data generated or analyzed during this study are included in this published article and its supplementary information files.

## Abbreviations

FLR: Future liver remnant
GRADE: Grading of recommendations assessment, development and evaluation
HCC: Hepatocellular carcinoma
MAFLD: Metabolic-associated fatty liver disease
MetS: Metabolic syndrome
NAFLD: Non-alcoholic fatty liver disease
NASH: Non-alcoholic steatohepatitis
OR: Odds ratio
OS: Overall survival
PHLF: Post-hepatectomy liver failure
QUIPS: Quality in Prognostic Studies
RFS: Recurrence-free survival
SAF score: steatosis, activity, fibrosis score
