# Supplementary material for: Is metabolic syndrome a risk factor in hepatectomy? A meta-analysis with subgroup analysis for histologically confirmed hepatic manifestations
Source: BMC Med. 2022 Jan 28;20:47. doi: 10.1186/s12916-022-02239-x (PMC8802506; doi:10.1186/s12916-022-02239-x)
Supplement: Supplementary file 1 — Additional file 1. Supplementary material. [file 12916_2022_2239_MOESM1_ESM.docx]

**Literature search:**

Population: patients undergoing hepatectomy

Risk factor and contol: patients with and without metabolic syndrome

Outcomes: complications, mortality and survival after hepatectomy

**Pubmed:**

((“non alcoholic”[tiab] OR “non-alcoholic”[tiab] OR nonalcoholic[tiab] OR metabolic[tiab]) AND fatty[tiab] AND liver[tiab]) OR NAFL*[tiab] OR ((“non alcoholic”[tiab] OR “non-alcoholic”[tiab] OR nonalcoholic[tiab] OR metabolic[tiab]) AND (steatohepatiti*[tiab] OR "steato hepatitis"[tiab] OR (hepatic[tiab] AND steatosis[tiab]))) OR NASH [tiab] OR MAFLD[tiab]

AND ("Hepatectomy"[Mesh] OR hemihepatectom*[tiab] OR posthepatectom*[tiab] OR post-hepatectom*[tiab] OR hepatectom*[tiab] OR ((surgery[tiab] OR surgeries[tiab] OR resection*[tiab]) AND liver[tiab]) OR sectionectom*[tiab] OR segmentectom*[tiab] OR „wedge resection“[tiab] OR "upper abdominal surgery"[tiab] OR "major abdominal surgery"[tiab] OR "Liver Transplantation"[Mesh])

OR "Non-alcoholic Fatty Liver Disease/surgery"[Mesh]

NOT (animals[mh] NOT humans[mh])

**Cochrane:**

((“non alcoholic” OR “non-alcoholic” OR nonalcoholic OR metabolic) AND fatty AND liver):ti,ab,kw

OR ((“non alcoholic” OR “non-alcoholic” OR nonalcoholic OR metabolic) AND (steatohepatiti* OR "steato hepatitis" OR (hepatic AND steatosis))) :ti,ab,kw

OR (NASH OR MAFLD OR NAFL*):ti,ab,kw

AND MeSH descriptor: [Hepatectomy] explode all trees

OR (hemihepatectom* OR posthepatectom* OR post-hepatectom* OR hepatectom*):ti,ab,kw

OR ((surgery OR surgeries OR resection*) AND liver):ti,ab,kw

OR (sectionectom* OR segmentectom* OR „wedge resection“ OR "upper abdominal surgery" OR "major abdominal surgery"):ti,ab,kw

OR MeSH descriptor: [Liver Transplantation] explode all trees

OR MeSH descriptor: [Non-alcoholic Fatty Liver Disease] explode all trees and with qualifier(s): [surgery - SU]

**Web of Science:**

TS = ((“non alcoholic” OR “non-alcoholic” OR nonalcoholic OR metabolic) AND fatty AND liver)

OR TS = ((“non alcoholic” OR “non-alcoholic” OR nonalcoholic OR metabolic) NEAR (steatohepatiti* OR "steato hepatitis" OR (hepatic AND steatosis)))

OR TS = (NASH OR MAFLD OR NAFL*)

AND TS = (hemihepatectom* OR posthepatectom* OR post-hepatectom* OR hepatectom*)

OR TS = ((surgery OR surgeries OR resection*) AND liver)

OR TS = (sectionectom* OR segmentectom* OR “wedge resection“ OR "upper abdominal surgery" OR "major abdominal surgery" OR transplantation)

NOT TI = (mice OR rats OR dogs OR animal OR murine OR rodent OR bovine OR porcine)
